# Safety and Immediate Efficacy of Pulmonary Thromboendarterectomy for Chronic Thromboembolic Disease

**DOI:** 10.1016/j.athoracsur.2025.05.037

**Published:** 2025-06-17

**Authors:** Elizabeth M. Bird, Jenny Z. Yang, Edward W. Mims, Kim M. Kerr, Demosthenes G. Papamatheakis, David S. Poch, Peter F. Fedullo, Atul Malhotra, Lori B. Daniels, Anna McDivit-Mizzell, Nicholas Phreaner, Nick H. Kim, Victor G. Pretorius, Michael M. Madani, Timothy M. Fernandes

**Affiliations:** 1 University of California San Diego School of Medicine, La Jolla, California; 2 Division of Pulmonary, Critical Care and Sleep Medicine, University of California San Diego, La Jolla, California; 3 Division of Pulmonary and Critical Care Medicine, University of Texas Southwestern, Dallas, Texas; 4 Division of Cardiovascular Medicine, University of California San Diego, La Jolla, California; 5 Division of Cardiovascular and Thoracic Surgery, University of California San Diego, La Jolla, California

## Abstract

**BACKGROUND:**

Chronic thromboembolic disease (CTED) is characterized by pulmonary vascular thromboembolic occlusions without elevation in pulmonary artery pressures or pulmonary vascular resistance at rest. Many patients have dyspnea on exertion despite normal resting hemodynamics and symptomatic improvement after pulmonary thromboendarterectomy surgery. We hypothesize that the safety and efficacy of pulmonary thromboendarterectomy in CTED will be similar to that in chronic thromboembolic pulmonary hypertension (CTEPH), which currently has a better characterized risk-benefit profile.

**METHODS:**

Patients who underwent pulmonary thromboendarterectomy for CTED from 2009 through 2022 had preoperative and postoperative pulmonary hemodynamics and postoperative course (n = 163) compared with a reference CTEPH cohort who underwent pulmonary thromboendarterectomy from 2017 to 2022 (n = 870). Preoperative rest hemodynamics were compared with both preoperative exercise and postoperative rest hemodynamics in patients with CTED who had measurements for all 3 conditions.

**RESULTS:**

The CTED cohort had 99 patients with complete preoperative rest, preoperative exercise, and postoperative hemodynamic measurements. Mean pulmonary artery pressure, pulmonary vascular resistance, and pulmonary artery compliance all changed abnormally with preoperative exercise but improved after surgery (21 [SD, 4], 36 [SD, 10], 18 [interquartile range {IQR}15–21] mm Hg; 175 [SD, 87], 205 [SD, 149], 126 [SD, 55] dyne·s·cm^−5^; 3.6 [IQR, 3.1–4.4], 2.7 [IQR, 2.1–3.6], 4.8 [IQR, 3.7–6.0] mL/mm Hg; preoperative rest, exercise, and postoperative for mean pulmonary artery pressure, pulmonary vascular resistance, and compliance; *P* < .001 for all comparisons, mean [SD] if normally distributed, otherwise median [IQR]). CTED patients had no in-hospital mortality and shorter hospital and intensive care unit lengths of stay (*P* < .001 for both) compared with the CTEPH cohort.

**CONCLUSIONS:**

Pulmonary thromboendarterectomy is safe and well-tolerated in patients with CTED, improving pulmonary hemodynamics and pulmonary artery compliance.

Many patients do not fully recover after a pulmonary embolism (PE), despite appropriate anticoagulation. Up to 50% of patients report worsened dyspnea compared with their pre-PE baseline,^1^ and perfusion defects fail to resolve in 30% to 50%.^2,3^ Chronic thromboembolic pulmonary hypertension (CTEPH) develops in ~2% to 3% of PE patients,^4^ but a more common condition, chronic thromboembolic disease (CTED), occurs in ~15%, causing exertional dyspnea without resting pulmonary hypertension.^2^

Pulmonary thromboendarterectomy (PTE) is the standard treatment for CTEPH, but CTED management remains controversial due to the lack of guidelines.^5^ Exercise hemodynamics are key to diagnosing CTED, with the slope of mean pulmonary artery pressure (mPAP) plotted against cardiac output (CO) helping to identify abnormal responses.^6–8^ Although small studies suggest PTE can improve symptoms and quality of life in CTED patients, major morbidity has been reported in up to 40% of cases.^9^

Pulmonary vascular resistance (PVR) is a key prognostic factor in CTEPH but remains normal at rest in CTED.^10^ Despite normal PVR at rest in CTED, right ventricular (RV) afterload may still be abnormal, because RV afterload is determined by pulmonary artery compliance (C_PA_) and impedance in addition to PVR.^11,12^ C_PA_ (calculated as the stroke volume divided by pulmonary artery pulse pressure) may be a better marker of CTED severity, and increases in C_PA_ after surgery may better capture improvement than PVR.^13,14^

This retrospective study assessed the safety and efficacy of PTE in CTED patients and evaluated preoperative and postoperative hemodynamics, focusing on changes in C_PA_.

## PATIENTS AND METHODS

All patients referred to University of California San Diego (UCSD) for PTE were reviewed by the pulmonary vascular medicine team. Eligible patients underwent evaluation and surgery. Data were obtained from UCSD’s quality improvement database (Institutional Review Board-exempt quality improvement database under project application 131373X). The database identified all patients who had PTE for CTED from March 2009 to December 2022. CTED patients were diagnosed by a multidisciplinary team based on the presence of exercise dyspnea and chronic pulmonary vascular occlusions without meeting the criteria for pulmonary hypertension at rest.

Pulmonary hypertension was defined using the 2019 criteria in Simonneau and colleagues,^7^ which requires patients to have PVR ≥240 dyne · s · cm^−5^, mPAP ≥20 mm Hg, and pulmonary artery wedge pressure (PAWP) <15 mm Hg. This CTED cohort was compared with an unmatched cohort of CTEPH patients who underwent PTE from January 2017 to December 2022. Patents were excluded from either cohort if on pulmonary hypertension medication before PTE. Major complications assessed included in-hospital death, extracorporeal membrane oxygenation, return to the operating room, bleeding, and reperfusion lung injury (new infiltrates in endarterectomized territories of lung with a PaO_2_-to-fraction of inspired oxygen ratio <150 within 72 hours of surgery and without other explanation.

Right heart catheterization was performed preoperatively to assess resting hemodynamics, including heart rate, mPAP, PAWP, and CO. A subgroup underwent exercise hemodynamics measurements using supine butterfly maneuvers with hand weights, with hemodynamics measured when a patient could no longer tolerate the weighted maneuvers. Postoperative hemodynamic variables were measured continuously using pulmonary artery catheter, with central venous pressure used as a surrogate for PAWP. Stroke volume, PVR, total pulmonary resistance, and C_PA_ were calculated using standard methods.

Preoperative and postoperative comparisons were made between CTED and CTEPH cohorts for demographics, hemodynamics, and complications. Continuous variables were tested for normality, with normal data presented as mean (SD) or median and interquartile range (IQR) and compared using *t* tests or nonparametric tests. Categorical data were assessed with χ^2^ or Fisher exact tests. Length of stay distributions were analyzed using the Kolmogorov-Smirnov test. A *P* value <.05 was considered significant. Statistical analysis was performed in MATLAB 2023a software (MathWorks).

## RESULTS

### PATIENT CHARACTERISTICS.

PTE was performed in 1654 patients (n = 870 CTEPH and n=163 CTED) at UCSD from January 2009 to December 2022 (Table 1). The CTED cohort was younger (47 vs 58 years, *P* < .001) and had higher body mass index (32 vs 29 kg/m^2^, *P* < .001) compared with CTEPH patients. Most CTED patients were New York Heart Association Functional Classification II to III (95%), with no class IV patients, compared with 7% in CTEPH (*P* < .001). Both groups predominantly had disease in level 2 or 3 in both lungs, although CTED patients had fewer level 1 cases. History of deep venous thrombosis was more common in CTEPH (58% vs 48%, *P* = .02).

### CTED VS CTEPH: PREOPERATIVE AND POSTOPERATIVE HEMODYNAMICS.

**FIGURE 1:**
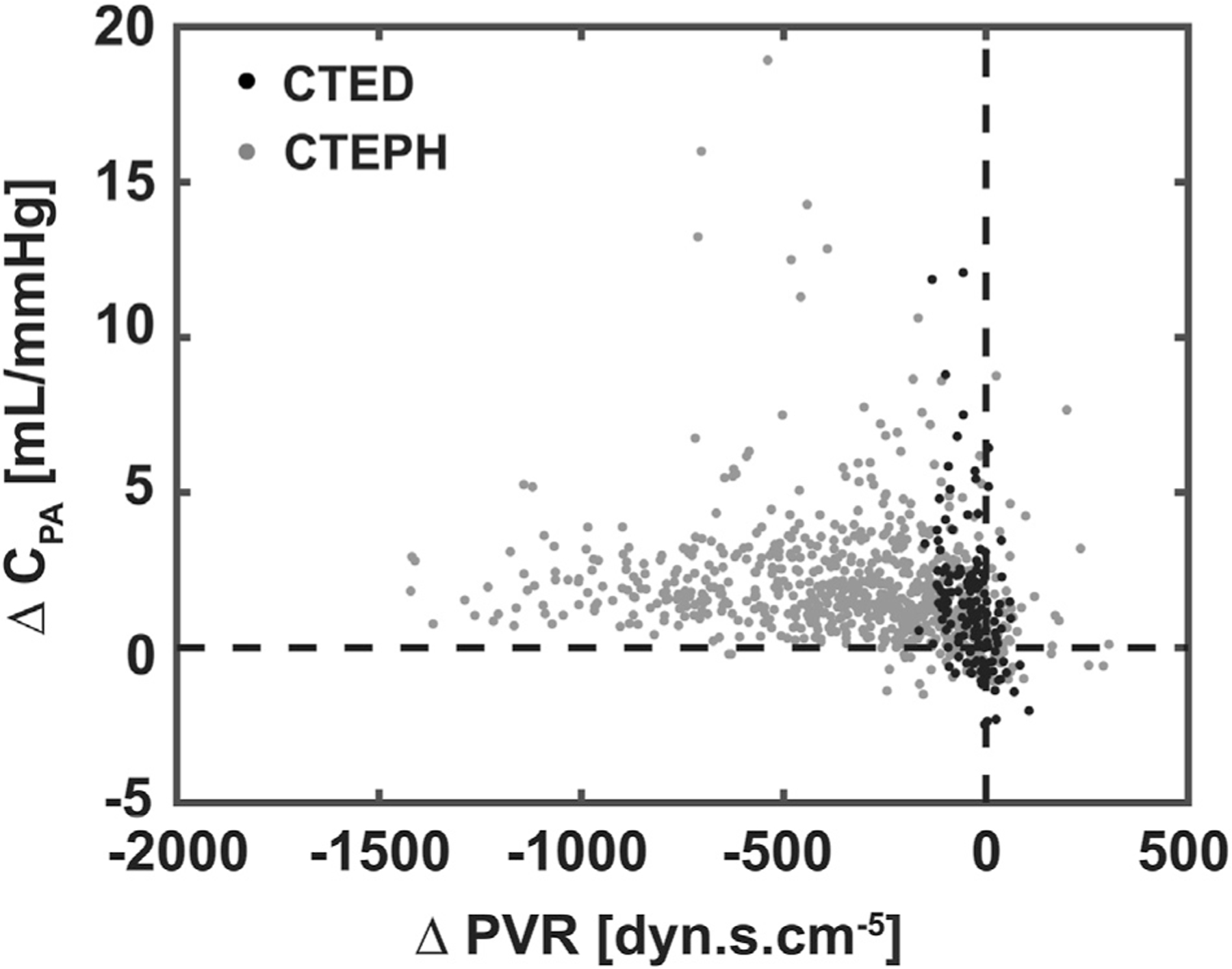
Change in pulmonary artery compliance (C_PA_) and pulmonary vascular resistance (PVR) after pulmonary thromboendarterectomy (PTE). Improvement in chronic thromboembolic disease (CTED) patients (white dots) is primarily through increased C_PA_, whereas in chronic thromboembolic pulmonary hypertension (CTEPH) patients (black dots) it is primarily through reduction of PVR, though there is also reduction in C_PA_.

Preoperative hemodynamics, except PAWP, showed significant differences between CTED and CTEPH patients (Table 2). CTED patients had lower mPAP (23 vs 42 mm Hg, *P* < .001) and PVR (179 vs 485 dyne · s · cm^−5^, *P* < .001). C_PA_ was higher in CTED patients (3.5 vs 1.4 mL/mm Hg, *P* < .001), whereas the resistance-compliance time constant was lower (0.44 vs 0.52 seconds, *P* < .001). After PTE, both groups showed improvement (Figure 1), but C_PA_ remained higher in CTED patients (4.7 vs 3.1 mL/mm Hg, *P* < .001).

### CTED: HEMODYNAMICS AT REST, EXERCISE, AND AFTER PTE.

**FIGURE 2:**
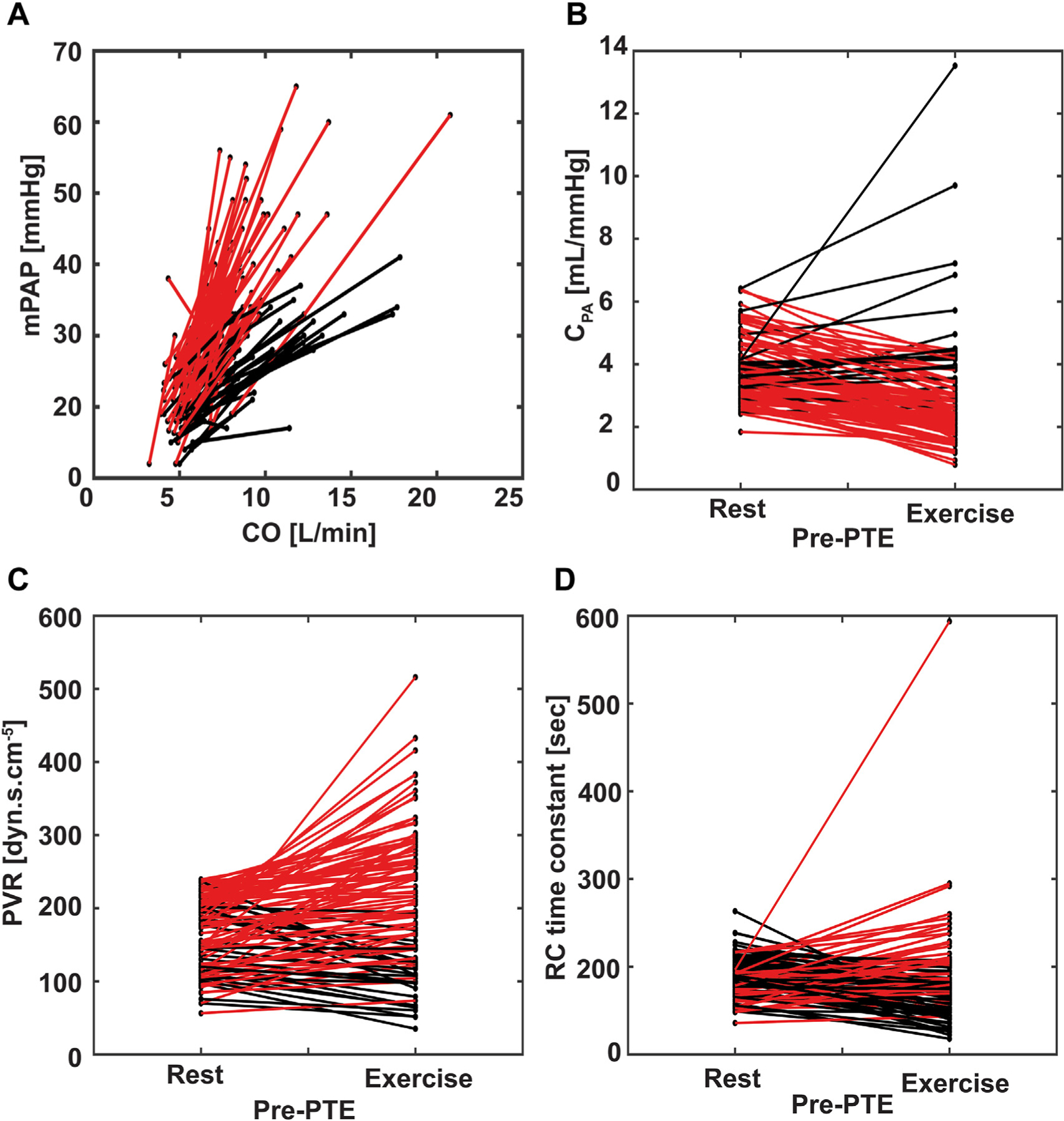
Preoperative hemodynamic changes with exercise in patients with chronic thromboembolic disease. Each line represents an individual patient. Some patients had abnormal changes in hemodynamics with exercise, plotted in red, whereas all others are plotted in black. (A) Slope of mean pulmonary artery pressure (mPAP)/cardiac output (CO) between rest and exercise. The slope was >3 mm Hg/min/L in 63% of patients. (B) Pulmonary artery compliance (C_PA_) at rest and exercise before pulmonary thromboendarterectomy (PTE). An abnormal decrease in C_PA_ occurred with exercise in 78% of patients. (C) Peripheral vascular resistance (PVR) at rest and exercise before PTE. An abnormal increase in PVR with exercise occurred in 74% of patients. (D) Resistance-compliance (RC) time constant at rest and exercise. An abnormal increase in RC time constant with exercise was documented in 41% of patients.

Among 99 CTED patients with complete pre-operative and postoperative data, mPAP increased significantly with exercise (21 vs 36 mm Hg, *P* < .001) (Table 3, Figure 2), with 69% of patients exceeding mPAP >30 mm Hg and 63% with a mPAP/CO slope >3 mmHg/min/L. PVR also increased during exercise (175 vs 205 dyne · s · cm^−5^, *P* < .001), with 74% of patients with a PVR increase and 40% with exercise PVR >240 dyne · s · cm^−5^. C_PA_ decreased with exertion (3.6 vs 2.7 mL/mm Hg, *P* < .001), affecting 78% of patients. After PTE, all hemodynamic measurements improved, with mPAP at 18 mm Hg, PVR at 126 dyne · s · cm^−5^, and C_PA_ at 4.8 mL/mm Hg (all *P* < .001).

### POSTOPERATIVE CHARACTERISTICS AND COMPLICATIONS.

No deaths occurred in the CTED group, compared with 14 deaths (1.6%, *P* = .14) in the CTEPH cohort (Table 4). No CTED patients required extracorporeal membrane oxygenation, whereas 1% of CTEPH patients did. Complications, such as postoperative bleeding, were similar (5% vs 9%, *P* = .12), and none of the CTED patients experienced intracranial hemorrhage. CTED patients had shorter circulatory arrest time (30 vs 44 minutes, *P* < .001), shorter intensive care unit and postoperative stays, and less time on mechanical ventilation. Additionally, fewer CTED patients were discharged home with oxygen (25% vs 63%, *P* < .001).

## COMMENT

Patients with CTED have chronic pulmonary vascular obstructions and exertional dyspnea despite lack of resting pulmonary hypertension. The large case series of CTED patients presented here shows that PTE can be performed safely in CTED patients, with minimal morbidity and mortality. The findings demonstrate that these patients have abnormal hemodynamics during exercise (increased PVR and decreased C_PA_), which PTE immediately improves (increased C_PA_ and reduced PVR at rest).

Our findings support that C_PA_ reflects CTED disease status. The C_PA_ values in our CTED cohort at rest and exercise were both lower than expected when considering normal values at exercise and rest (C_PA_ at rest ranges from 4 to 12 mL/mm Hg and usually remains >4 mL/mm Hg with exercise).^15–17^ In contrast, PVR remains within the normal range for all CTED patients at rest and for most with exercise. The observed C_PA_ decrease with exercise relative to rest was consistent with that in other published cohorts and may also complement other hemodynamic measures in identifying CTED.^18–21^ In our study, decrease in C_PA_ with exercise was one of the most sensitive markers for abnormal exercise hemodynamics, with 78% of patients having a decrease in C_PA_ with exertion.

Applying the previous definition of exercise-induced pulmonary hypertension (mPAP >30 mm Hg with exercise) to our CTED cohort, only 69% would have met the exercise-induced pulmonary hypertension criterion despite all having symptomatic disease.^22^ Other proposed measures to identify CTED from exercise-induced hypertension include an mPAP/CO slope value of >3 mm Hg/min/L.^5,6,23^ With this threshold, only 63% of our patients would have been correctly classified. Using an increase in PVR with exercise to identify exercise-induced pulmonary hypertension would correctly classify 74% of CTED patients.

Unfortunately, PVR and PAWP measures can be difficult to obtain with exercise (ie, there is an inherent delay between measurement of CO and PAWP). Because patients with CTED have, by definition low resting PVR, the RV afterload (a function of PVR and C_PA_) seen in these patients may be primarily due to C_PA_.^11^ This notion is consistent with our findings of C_PA_ being more sensitive than the mPAP/CO slope at detecting abnormal exercise hemodynamics in patients with PVR in the range of normal (ie, <240 dyne · s · cm^−5^), and may explain why C_PA_ captures patients with abnormal pulmonary hemodynamic response to exercise, despite mPAP <30 mm Hg with exercise.

Our findings suggest that PTE at an experienced center is both safe and effective in CTED. In this CTED cohort, PTE resulted in an immediate improvement in RV afterload, with postoperative C_PA_ improved to the lower estimates of normal seen in studies of healthy controls.^15^ Although we did not design our study to compare PTE and balloon pulmonary angioplasty outcomes, a small initial CTED cohort suggests balloon pulmonary angioplasty may also similarly improve C_PA_ from below normal to values near the lower limit of normal.^24^ A more in-depth understanding of the differences between PTE and balloon pulmonary angioplasty requires further investigation.

Similarly, our study design does not allow us to compare post-PTE outcomes with nonoperative outcomes or identify CTED patients who are poor PTE candidates. Although patients with CTED who do not undergo PTE typically have stable pulmonary hemodynamics over years without disease progression, their symptoms, functional limitations (6-minute walk test, New York Heart Association Functional Classification), and disease impact on quality of life are also significant and without improvement.^25^ All CTED patients at UCSD undergo multidisciplinary review for PTE consideration, but specific studies informing the risk-benefit considerations for CTED patients are still needed. This study contributes to this need, demonstrating the safety of PTE in CTED patients.

Although not directly assessed in our study, the PTE-driven improvement in C_PA_ may also be associated with improvement in exercise capacity after PTE.^12,14^ de Perrot and colleagues^14^ found that although only 25% of CTEPH patients with postoperative C_PA_ <2.0 mL/mm Hg improved to New York Heart Association Functional Classification I, >90% of CTEPH patients with postoperative C_PA_ >4.0 mL/mm Hg improved to functional class I. After PTE, most of our CTED cohort had C_PA_ >4.0, with 1 patient having C_PA_ <2.0. Future work requires formal evaluation of post PTE functional capacity in our CTED cohort to confirm the correlation with C_PA_ improvement.

Full CPET testing with ventilatory dead space and minute ventilation/ventilatory equivalents of carbon dioxide (VE/Vco_2_) slope measurement would also improve our ability to interpret PTE efficacy on functional capacity changes in CTED. Dead space ventilation and the VE/Vco_2_ slope have both been correlated with symptoms in CTED and with outcomes in CTEPH, and other centers have shown that surgical removal of these organized clots and the immediate improvement in hemodynamics translate to improved long-term outcomes, including VE/Vco_2_, functional status and quality of life.^9,26–29^ CPET would have also improved standardization of exercise pulmonary hemodynamic measurement. Unfortunately, CPET was not routinely done until more recently at our institution, so given the paucity of data in this cohort, it was excluded from this analysis.

Some exercise testing limitations affected our cohort. First, exercise testing was performed using butterfly maneuvers, which may have resulted in submaximal exercise. However, this form of exercise still achieved the goal of increasing heart rate and CO, before repeating hemodynamic measurements.

Additionally, postoperative exercise hemodynamic variables were not measured, limiting the evaluation of PTE’s effects on the normalization of C_PA_ changes with exercise. Specifically, a postoperative abnormal decrease in C_PA_ with exercise has been associated with persistent functional limitation despite successful PTE (post-PTE normalization of PVR and C_PA_ at rest) in CTEPH patients.^13^ Furthermore, post-PTE functional improvement in CTED patients has occurred in a cohort that had improvement in only postoperative exercise C_PA_ and not C_PA_ at rest.^29^ Although it is possible that no improvement was seen in C_PA_ at rest because patients had rest C_PA_ close to the lower limit of normal both preoperatively and postoperatively (3.9 and 4.0 mL/mm Hg, respectively) functional improvement after PTE.^29^ Further work will include analysis of both rest and exercise post-PTE hemodynamics.

The evolving classification criteria for CTED present another limitation for this study. For patient classification and data analysis, the most recent CTEPH classification criteria (mPAP >20 mm Hg and PVR >240 dyne · s · cm^−5^; Simonneau and colleagues^7^) relevant to our study cohort spanning 2009 to 2022 was applied to our analysis. Given guidelines before 2019 required patients to have mPAP >25 mm Hg to be classified as CTEPH, there are patients in our cohort with mPAP >20 mmHg (but <25 mm Hg) who were classified as having CTEPH at the time of diagnosis and therefore did not undergo exercise testing. By the time this work is published, there will be at least 1 more recently updated set of guidelines that change the definition of CTEPH to include those with PVR >160 dyne · s · cm^−5^ (Simonneau and colleagues, 2023).^30^ Although out of the scope of our current study, the hemodynamic effects of PTE in patients with CTED using the 2023 criteria vs CTEPH previously classified as CTED (PVR 2 to 3 dyne · s · cm^−5^ and mPAP >20 mmHg) should be compared. Because the CTED group had no deaths, reclassification of CTED using the 2023 criteria would not increase CTED mortality rates, supporting PTE safety for patients with either normal resting hemodynamics or milder resting pulmonary hypertension.

Selection bias in those chosen for PTE and the lack of long-term follow-up are additional limitations of our study. Patients who were invited for PTE evaluation for CTED tended to have few comorbidities and were younger than the CTEPH cohort. There may be patients with CTED who are older and with more comorbidities who are never referred for evaluation by their physicians or who are not considered appropriate surgical candidates.

Additionally, UCSD operates as a large referral center for CTEPH. Most of our patients are not local, which hinders our ability for return visits and postoperative assessment. For long-term follow-up data, we rely on our colleagues from centers in countries with nationalized medical systems that allow for single-center long-term follow-up.

Another limitation is the inclusion of patients from 2009–2016 in our CTED but not CTEPH cohort to capture as much PTE outcome data as possible in the significantly smaller CTED population. There have been no changes in surgeons performing PTE or major changes to surgical technique, which should limit the variability introduced from these earlier cases.

Finally, it should be noted that some assumptions are included in the commonly used calculation of C_PA_, related to linearity and mechanical properties of the vessel.^31,32^ Although we acknowledge this metric as imperfect, it nonetheless provides a useful ratio which is clinically accessible and has been used in multiple other studies.^13,28^

Our findings suggest that C_PA_ is reduced at rest and has an abnormal decrease in response to exercise in patients with CTED. PTE improves C_PA_ at rest, supporting the effectiveness of PTE as a treatment for patients with CTED and normal or nearly normal resting hemodynamics. PTE is safe in patients with CTED, with low postoperative morbidity, similar to CTEPH, and no postoperative deaths. Further work could characterize the heterogeneity of exercise hemodynamics in CTED patients and the associations of postoperative rest vs exercise hemodynamics with patient functional improvement.

## Figures and Tables

**TABLE 1:** CTED vs CTEPH: Baseline Patient Characteristics

Variable	CTED (n = 163)	CTEPH (n = 870)	*P* Value
Age, y	47 (35–62)	58 (43–67)	<.001
Body mass index, kg/m^2^	32 (27–37)	29 (25–34)	<.001
Male sex	75 (46)	464 (53)	.08
NYHA Functional Classification^a^			<.001
I	8 (5)	9 (1)	
II	57 (35)	163 (19)	
III	98 (60)	635 (73)	
IV	0 (0)	60 (7)	
Disease level			<.001
Right lung			
0	4 (3)	3 (0)	
1	15 (12)	250 (29)	
2	68 (52)	341 (39)	
3	35 (27)	228 (26)	
4	8 (6)	48 (6)	
Left lung			<.001
0	14 (11)	19 (2)	
1	7 (5)	112 (13)	
2	57 (44)	340 (39)	
3	45 (35)	301 (35)	
4	7 (5)	98 (11)	
Medical history			
Deep venous thrombosis	79 (48)	508 (58)	.02
Pulmonary embolism	156 (96)	839 (96)	.65
Splenectomy	8 (5)	37 (4)	.71
Family history of VTE	42 (26)	216 (25)	.8

a Functional class at time of immediate preoperative evaluation.

Data are presented as n (%) or median (interquartile range). CTED, chronic thromboembolic disease; CTEPH, chronic thromboembolic pulmonary hypertension; NYHA, New York Heart Association; VTE, venous thromboembolism.

**TABLE 2:** CTED vs CTEPH: Preoperative and Postoperative Hemodynamics

Variable	CTED (n = 163)	CTEPH (n = 870)	*P* Value
Preoperative			
Heart rate, beats/min	67 (61–77)	72 (63–80)	.002
Right atrium, mm Hg	7 (5–9)	9 (6–12)	<.001
PAP systolic, mm Hg	39 (32–46)	72 (58–85)	<.001
PAP diastolic, mm Hg	14 (12–18)	25 (19–31)	<.001
Mean PAP, mm Hg^a^	23 (19–27)	42 (33–49)	<.001
PAWP, mm Hg	11 (8.3–14)	11 (9–14)	.45
PVR, dyn · s · cm^−5^	179 (135–212)	485 (320–748)	<.001
Total PVR, dyn · s · cm^−5^	332 (108)	691 (448)	<.001
Cardiac output, L/min^a^	6 (5–6)	5 (4–6)	<.001
Cardiac index, L/min/m^2^	2.8 (2.4–3.1)	2.3 (1.9–2.7)	<.001
C_PA_, mL/mm Hg	3.5 (2.9–4.1)	1.4 (1–2.1)	<.001
Resistance-compliance time, s	0.44 (0.14)	0.52 (0.16)	<.001
Postoperative			
Heart rate, beats/min	83 (78–92)	80 (72–87)	<.001
Central venous pressure, mm Hg	8 (6–11)	8 (5–10)	.15
PAP systolic, mm Hg	27 (23–33)	37 (31–44)	<.001
PAP diastolic, mm Hg	12 (5)	14 (6)	<.001
Mean PAP, mm Hg	19 (15–21)	22 (19–27)	<.001
PVR, dyn · s · cm^−5^	131 (56)	204 (125)	<.001
TPR, dyn · s · cm^−5^	237 (196–307)	320 (258–404)	<.001
Cardiac output, L/min	5.9 (5.3–6.9)	5.5 (4.8–6.3)	<.001
Cardiac index , L/min/m^2^	2.8 (2.5–3.3)	2.7 (2.4–3.1)	.01
C_PA_, mL/mm Hg	4.7 (3.8–6)	3.1 (2.4–4.2)	<.001
Resistance-compliance time, s	0.45 (0.37–0.57)	0.48 (0.4–0.58)	.08

a Mean PAP and cardiac output slope were only calculated in CTED patients with exercise data.

Data are presented as median (interquartile range) or as mean (SD). C_PA_, pulmonary artery compliance; CTED, chronic thromboembolic disease; CTEPH, chronic thromboembolic pulmonary hypertension; PAP, pulmonary artery pressure; PAWP, pulmonary artery wedge pressure; PVR, peripheral vascular resistance; TPR, total pulmonary resistance.

**TABLE 3:** Chronic Thromboembolic Disease Hemodynamics: Preoperative at Rest and Exercise and Postoperative at Rest

Variable	Pre-PTE (n = 99)	Exercise (n = 99)	*P* Value^a^	Post-PTE (n = 99)	*P* Value^b^
Heart rate, beats/min	68 (61–77)	100 (88–118)	<.001	84 (15)	<.001
PAP systolic, mm Hg	36 (30–41)	55 (45–69)	<.001	27 (23–32)	<.001
PAP diastolic, mm Hg	13 (4)	21±(8)	<.001	12 (3)	.014
Mean PAP, mm Hg	21 (4)	36 (10)	<.001	18 (15–21)	<.001
PAWP, mm Hg	10 (8–13)	13 (10–15)	<.001	8 (6–10)^c^	—
PVR, dyn · s · cm^−5^	175 (87)	205 (149)	<.001	126 (55)	<.001
TPR, dyn · s · cm^−5^	306 (265–368)	310 (244–412)	.751	226 (194–314)	<.001
Cardiac output (L/min)	5.4 (4.9–6)	8.6 (7.8–10.4)	<.001	5.7 (4.9–6.9)	.006
Cardiac index, L/min/m^2^	2.7 (2.4–2.9)	4.4 (3.9–5.1)	<.001	2.9 (2.4–3.3)	.009
C_PA_ (mL/mmHg)	3.6 (3.1–4.4)	2.7 (2.1–3.6)	<.001	4.8 (3.7–6)	<.001
RC time, ms	0.44 (0.37–0.51)	0.4 (0.29–0.52)	.098	0.44 (0.35–0.57)	.085
mPAP/cardiac output slope, mm Hg/min/L		3.9 (2.0–6.2)	—		

a Comparison of pre-PTE vs exercise

b Comparison of pre-PTE vs post-PTE

c Postoperative PAWP is estimated as the central venous pressure.

Data are presented as median (interquartile range) or mean (SD). C_PA_, pulmonary artery compliance; PAP, pulmonary artery pressure; PAWP, pulmonary artery wedge pressure PTE, pulmonary thromboendarterectomy; PVR, peripheral vascular resistance; RC, resistance-compliance; TPR total pulmonary resistance.

**TABLE 4:** CTED vs CTEPH: Operative Characteristics and Postoperative Complications

Variable	CTED	CTEPH	*P* Value
Intraoperative characteristics	(n = 163)	(n = 870)	
Concurrent operation:			
Coronary artery bypass grafting	7 (4)	63 (7)	.23
Patent foramen ovale closure	30 (18)	161 (19)	1
Valve repair	2 (1)	16 (2)	.75
Total circulatory arrest time, min	30 (20–39)	44 (34–56)	<.001
Postoperative characteristics			
Postoperative time on ventilator, d	1 (1–1)	1 (1–2)	<.001
Length of stay			
Intensive care unit, d	2 (2–4)	4 (3–6)	<.001
Postoperative, d	9 (7–11)	10 (8–13)	<.001
Postoperative complications			
Deaths before discharge	0 (0)	14 (2)	.14
Extracorporeal membrane oxygenation	0 (0)	9 (1)	.37
Return to operating room	3 (2)	25 (3)	.6
Bleeding	8 (5)	76 (9)	.12
Reperfusion lung injury	9 (6)	92 (11)	.04
Atrial arrhythmia	36 (22)	256 (29)	.06
Hemidiaphragm paresis	4 (2)	45 (5)	.16
Pericardial effusion	33 (20)	184 (21)	.83
Postoperative thrombosis	3 (2)	30 (3)	.46
Pneumonia/tracheobronchitis	5 (3)	63 (7)	.06
Discharged with oxygen	41 (25)	551 (63)	<.001

Data are presented as n (%) or median (interquartile range). CTED, chronic thromboembolic disease; CTEPH, chronic thromboembolic pulmonary hypertension.

## Abbreviations and Acronyms

C_PA_: pulmonary artery compliance
CO: cardiac output
CTED: chronic thromboembolic disease
CTEPH: chronic thromboembolic pulmonary hypertension
mPAP: mean pulmonary artery pressure
PAWP: pulmonary artery wedge pressure
PE: pulmonary embolism
PTE: pulmonary thromboendarterectomy
PVR: pulmonary vascular resistance
RC: resistance-compliance
RV: right ventricle
UCSD: University of California San Diego
Vco_2_: ventilatory equivalents of carbon dioxide
VE: minute ventilation
